# SOCS3 Silencing Attenuates Eosinophil Functions in Asthma Patients

**DOI:** 10.3390/ijms16035434

**Published:** 2015-03-10

**Authors:** Mª Paz Zafra, Jose A. Cañas, Carla Mazzeo, Cristina Gámez, Veronica Sanz, Mar Fernández-Nieto, Santiago Quirce, Pilar Barranco, Javier Ruiz-Hornillos, Joaquín Sastre, Victoria del Pozo

**Affiliations:** 1 Department of Immunology, IIS-Fundación Jiménez Díaz, 28040 Madrid, Spain; E-Mails: mp_zafra@hotmail.es (M.P.Z.); jose.canas@fjd.es (J.A.C.); cmazzeo@fjd.es (C.M.); cgamez@fjd.es (C.G.); vsanz@fjd.es (V.S.); 2 Centro de Investigación Biomedica En Red de Enfermedades Respiratorias (CIBERES), 28029 Madrid, Spain; E-Mails: mmfernandez@fjd.es (M.F.-N.); squirce@gmail.com (S.Q.); mpbarranco@gmail.com (P.B.); jsastre@fjd.es (J.S.); 3 Department of Allergy, Fundación Jiménez Díaz, 28040 Madrid, Spain; 4 Department of Allergy, Hospital La Paz Health Research Institute (IdiPAZ), 28046 Madrid, Spain; 5 Department of Allergy, Hospital Infanta Elena, Valdemoro, 28342 Madrid, Spain; E-Mail: javier.ruiz@idcsalud.es

**Keywords:** short interfering RNAs (siRNAs), SOCS3, eosinophils, asthma

## Abstract

Eosinophils are one of the key inflammatory cells in asthma. Eosinophils can exert a wide variety of actions through expression and secretion of multiple molecules. Previously, we have demonstrated that eosinophils purified from peripheral blood from asthma patients express high levels of suppressor of cytokine signaling 3 (SOCS3). In this article, *SOCS3* gene silencing in eosinophils from asthmatics has been carried out to achieve a better understanding of the suppressor function in eosinophils. SOCS3 siRNA treatment drastically reduced SOCS3 expression in eosinophils, leading to an inhibition of the regulatory transcription factors GATA-3 and FoxP3, also interleukin (IL)-10; in turn, an increased STAT3 phosphorilation was observed. Moreover, SOCS3 abrogation in eosinophils produced impaired migration, adhesion and degranulation. Therefore, SOCS3 might be regarded as an important regulator implicated in eosinophil mobilization from the bone marrow to the lungs during the asthmatic process.

## 1. Introduction

Asthma is a complex chronic disease of the airways classified into several different inflammatory phenotypes. The most prominent of these phenotypes is Th2-driven inflammation, and approximately half of all patients with asthma seem to belong to this phenotype, which is mainly associated with eosinophilic inflammation of the predominant tissue [1].

Eosinophils are terminally differentiated non-dividing cells, and infiltrate tissue from blood circulation. Therefore, these cells have to migrate from the bloodstream to inflamed airways after be activated and recruited by chemoattractants, such as eotaxins and interleukin (IL)-5 [2]. First, they start to roll along the endothelium to establish a solid adhesion on the endothelium due to the interaction of the integrins on eosinophils with adhesion molecules expressed in endothelial cells, and then eosinophils extravasate to the target tissue to exert their functions [3]. Eosinophils have traditionally been described as effector cells in asthma, as they have the capacity to release an array of mediators that damage the epithelium and induce mucus production and bronchoconstriction; however, recent studies have revealed more diverse roles for eosinophils, displaying a great variety of immunoregulatory actions. Thus, eosinophils can act as professional antigen presenting cells and can modulate T CD4^+^ cells, dendritic cells, B cells, mast cells, neutrophils, and basophils [4]. In this way, eosinophils express a specific cytokine profile to carry out the regulation and polarization of these cells towards a new functional subtype; in allergic asthma, where Th2 responses are predominant, IL-4, IL-5, and IL-13 are the principal cytokines secreted [2,5]. These cytokines bind to membrane receptors to activate complex signal transduction pathways.

Cytokine function is strictly controlled by the so-called suppressor of cytokine signaling (SOCS) family proteins to prevent an imbalance in the magnitude, duration, and remission of the immune response. The cytokine induced STAT-inhibitor (CIS)-SOCS family is comprised of eight members [6]. SOCS is a family of molecules that suppress the JAK-STAT signaling pathway and regulate Th cell differentiation. SOCS3 is an inhibitor that is relatively specific to STAT3; in addition, it can inhibit other signaling pathways, such as Ras/ERK and PI3K, which affects cell proliferation, survival, and differentiation [7,8]. In the past several studies have reported a selective expression of SOCS3 in allergic type Th2 cells [9,10], and also in eosinophils [11]. SOCS3 expression is enhanced by Th2 cytokines in eosinophils; and, in T-cells, SOCS3 levels correlated with disease severity and IgE levels in asthmatic patients [12].

The cyclooxigenase product, prostaglandin E_2_ (PGE_2_), is produced during inflammatory responses. Our group has demonstrated that it is present in lungs from subjects with asthma [13], and is capable of inducing and regulating SOCS3 expression in human eosinophils [11].

Gene silencing by siRNA (short interfering RNA) has a well-established role as a tool for basic research in biology to investigate the potential of new protein targets and further validate their function and the mechanisms through which they act. Using different *in vitro* and *in vivo* techniques, we recently demonstrated that SOCS3-siRNA intranasal delivered in a mouse model of chronic allergic asthma leads to inhibition of the asthmatic response [14]. Therefore, siRNA technology has been used again to better understand the role of SOCS3 in human eosinophils, as central players in asthma pathogenesis.

## 2. Results

### 2.1. SOCS3 Down-Regulation in Blood Eosinophils from Asthmatic Patients by siRNA

**Figure 1:**
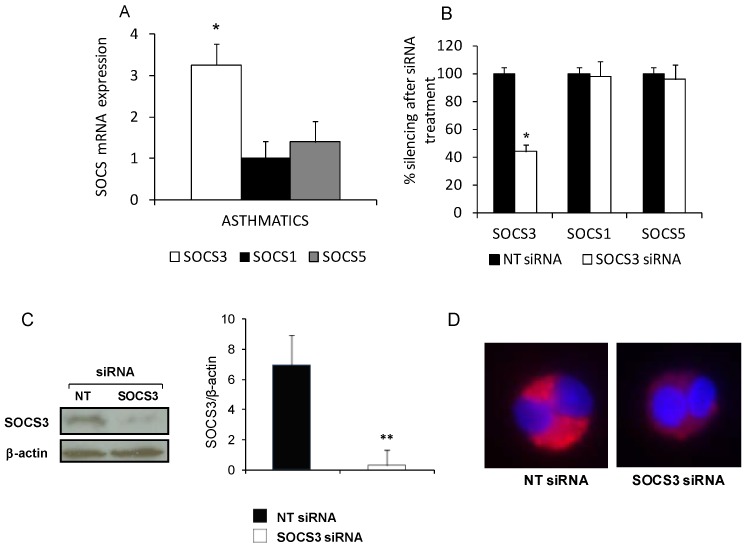
siRNA technology reduces suppressor of cytokine signaling 3 (SOCS3) expression in eosinophils from asthmatic patients. (**A**) SOCS3, SOCS1 and SOCS5 mRNA relative levels were measured by quantitative PCR in eosinophils purified from asthmatic patients (*n* = 36); (**B**) After SOCS3 siRNA incubation, quantitative-PCR results showed significant down-regulation of SOCS3 mRNA levels and no alteration of SOCS5 or SOCS1, as well as decreased protein expression level of SOCS3 after SOCS3 siRNA treatment was detected by Western blot analysis and further quantified by densitometry (**C**) or immnunofluoresce (**D**) as is depicted on a representative picture of an eosinophil incubated with non-target (NT)siRNA (**left** panel) or SOCS3 siRNA (**right** panel), after staining with anti-SOCS3 antibody labeled with Alexa 647 fluorochrome. The results were expressed as mean ± SD, (*n* = 6–8). * Denotes statistical significance (*p* < 0.05); ** denotes obvious statistical significance (*p* < 0.01).

Initially, SOCS3, SOCS1 and SOCS5 gene expression in purified blood eosinophils from asthmatics was determined. SOCS3 mRNA levels were found increased when compared with SOCS1 and SOCS5 transcripts abundance (Figure 1A). Hence, SOCS3 has been targeted by siRNA technology to downregulate its expression.

We performed assays to determine whether naked SOCS3 siRNAs were able to produce SOCS3 down-regulation in purified eosinophils. In order to quantify SOCS3 reduction, we evaluated *SOCS3* gene expression and encoded protein in eosinophils by quantitative PCR and Western blotting after SOCS3-silencing. A 56% reduction in mRNA relative levels was detected in those eosinophils, which had received SOCS3 siRNA in comparison with eosinophils cultured with the non-target (NT) siRNA (*p* < 0.01, Figure 1B). Protein evaluation revealed a marked decrease in SOCS3 expression as a consequence of the siRNA specific effect exerted in eosinophils (*p <* 0.001, Figure 1C).

Furthermore, to corroborate SOCS3 siRNA specificity, SOCS5 and SOCS1 relative gene expressions in eosinophils from asthmatic donors were also assessed by qPCR (Figure 1B). As expected, none of them showed a significant variation in their mRNA relative levels due to the SOCS3 siRNA.

As shown in Figure 1D, SOCS3 expression intensity within the eosinophil cytoplasm, measured as red fluorescence detected by confocal microscopy in response to the 594 nm laser beam, was attenuated after SOCS3 siRNA treatment; while eosinophils incubated with the negative silence control displayed a strong red fluorescence in granules, demonstrating that the higher SOCS3 expression found in asthmatic patients can be down-regulated using naked siRNA.

It is important to underline that after 48 h of eosinophil culture with SOCS3 siRNA or NT siRNA, the viability, assessed by tripan blue staining, did not vary significantly (data not shown).

### 2.2. Immune Response Elements Altered by Interfering SOCS3 Expression in Eosinophils from Asthmatic Subjects

We wanted to assess whether SOCS3 down-regulation modified the immune response profile expressed in blood eosinophils from asthmatic patients. So, GATA3, T-bet, and FoxP3, which are master transcription factors of Th2, Th1, and Treg polarization, respectively, were measured by qPCR.

**Figure 2:**
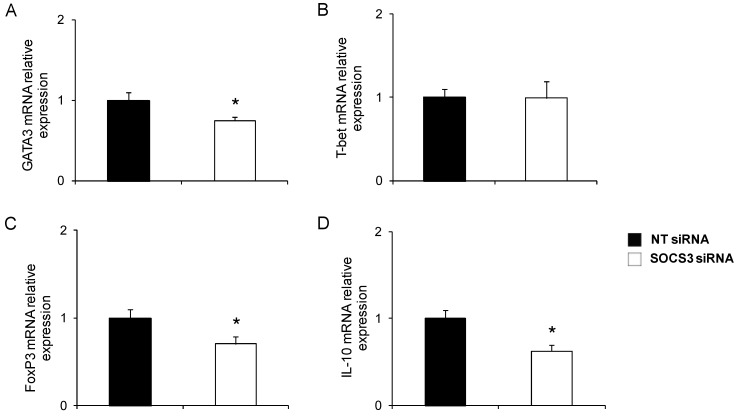
Phenotype evaluation in SOCS3-deficient eosinophils purified from asthma patients. GATA3 (**A**); T-bet (**B**); FoxP3 (**C**); and IL-10 (**D**) mRNA relative levels were determined by qPCR in blood eosinophils purified from five asthma patients treated with SOCS3 siRNA or NT siRNA. * *p <* 0.05 between groups.

Following 48 h of incubation in medium containing SOCS3 siRNA or NT siRNA, the analysis revealed a reduced transcription of GATA3 in eosinophils from patients with asthma (*n* = 5) treated with SOCS3 siRNA, but not in those incubated with the negative control (*p <* 0.05, Figure 2A). However, T-bet gene expression does not significantly vary in response to the SOCS3 expression reduction in eosinophils (Figure 2B).

Next, the effect of SOCS3 silencing on the regulatory immune response was analyzed. Measurement of FoxP3 in eosinophils from asthmatic volunteers (*n* = 5) revealed a significant reduction in Foxp3 gene expression compared to those treated with non-target siRNA (*p* < 0.05, Figure 2C). Concomitantly, IL-10 mRNA production was significantly diminished in response to SOCS3 interference (*p <* 0.05, Figure 2D).

### 2.3. Eosinophil Migration Suppressed by SOCS3 Gene Silencing

**Figure 3:**
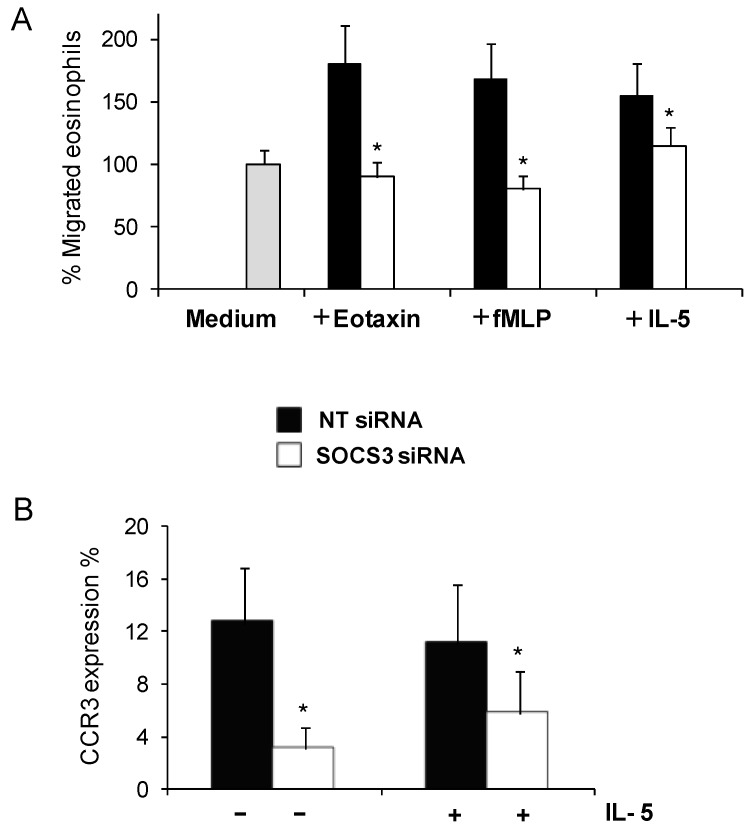
Reduced eosinophil migration due to SOCS3 down-regulation by gene silencing. Cell migration was detected by a transwell assay; percentages of eosinophils migrated towards IL-5, eotaxin and fMLP (**A**) were measured by flow cytometry after *SOCS3* silencing; and (**B**) CCR3 surface expression on eosinophils was achieved by the measure of FITC positive cells by flow cytometry. The results were expressed as mean ± SD, (*n* = 6–8). * Denotes statistical significance (*p* < 0.05) between groups.

After gene-silencing treatment, migration was checked in eosinophils from asthmatic patients. We have previously proved that IL-5 produces a drastic SOCS3 induction in eosinophils [11], so in the first instance IL-5 was used to stimulate eosinophil migration. Compared to NT siRNA treatment, SOCS3 siRNA treatment decreased eosinophil migration (73%) in response to IL-5 (Figure 3A, *p <* 0.05, *n* = 6). We have further tested eosinophils migration with eotaxin (CCL11), which is the most potent and specific chemoattractant of eosinophils, as well as with fMLP, a well-known inductor of eosinophil migration and activation (Figure 3A). We found that eosinophils cultured with SOCS3 siRNA exhibited a complete inhibition towards eotaxin and fMLP stimuli in comparison with the siRNA NT treatment (100%, *p <* 0.05, *n* = 3), reaching similar levels to basal eosinophil migration without stimuli (medium).

In order to determine the mechanism by which SOCS3 is directly implicated in migration inhibition, expression of eotaxin receptor levels by flow cytometry were measured. We observed that in eosinophils from asthmatic subjects treated with SOCS3 siRNAs, CCR3 expression was significantly reduced, with or without IL-5 stimulation (52% and 76%, respectively; *p <* 0.05, Figure 3B). These data indicate that the IL-5 stimulus, used to create a directed and more specific migration, did not substantially alter CCR3 expression.

### 2.4. SOCS3 Gene Silencing Reduced Eosinophil Adhesion

**Figure 4:**
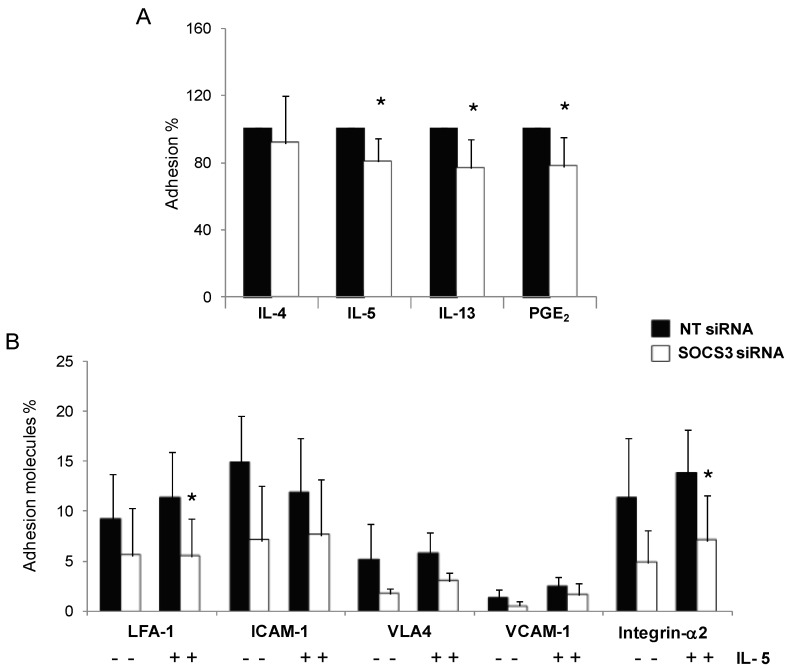
Impaired adhesion capacity in blood eosinophils from asthmatic patients after *SOCS3* gene silencing. (**A**) NT and SOCS3 siRNA treated eosinophils were incubated with IL-4, IL-5, IL-13, and PGE_2_ for 1 or 2 h and then allowed to adhere to fibronectin-coated wells for 30 min. Results are expressed as mean ± SD of adhered cell percentages estimated by residual eosinophils peroxidase (EPO) measurement; and (**B**) LFA-1, ICAM-1, VLA4, VCAM-1, and Integrin-α2 surface expression on blood eosinophils from seven asthmatic patients incubated with SOCS3 siRNA or NT siRNA were determined by flow cytometry. * *p <* 0.05 between groups.

To determine whether eosinophil *SOCS3* silencing produces changes in adhesion function, we compared adhesion of eosinophils from asthmatic patients treated with SOCS3 siRNA or scrambled siRNA over 48 h, after stimulation with different Th2 cytokines (IL-4, IL-5, and IL-13) or PGE_2_. Eosinophil adhesion was significantly reduced when they were cultured with IL-5, IL-13, or PGE_2_. These inhibitions observed were about 25% for IL-5, IL-13, and PGE_2_ (*p <* 0.05, Figure 4A). Stimulation with IL-4 did not produce any difference between SOCS3 silenced eosinophils and control eosinophils.

To determine which adhesion molecules were contributing to the effect of *SOCS3* silencing in eosinophil adhesion, the main cell adhesion molecules implicated in the process by flow cytometry were analyzed. Cells were stimulated with IL-5 or not, followed by staining with ICAM1/LFA1, VCAM1/VLA4, and CD49d. A general tendency toward decreased expression of all the markers tested was achieved in SOCS3 siRNA-treated eosinophils; however, only the reduction obtained after *SOCS3* silencing in LFA-1 and integrin-α2 expression reached statistical significance in comparison with control-treated eosinophils (53% and 49%, respectively, *p <* 0.05, Figure 4B), both conditions being IL-5 stimulated.

### 2.5. Eosinophil Degranulation Recovery after SOCS3 Silencing

**Figure 5:**
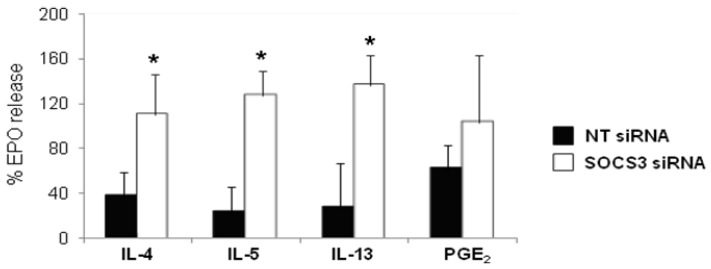
Augmented eosinophil degranulation in asthmatic blood eosinophils treated with SOCS3 siRNA. Purified blood eosinophils from eight asthmatic patients incubated with SOCS3 siRNA (white bars, mean ± SD) or NT siRNA (black bars, mean ± SD) for 48 h, were pretreated with IL-4, IL-5, IL-13 and PGE_2_ for 1 or 2 h and them stimulated with C5a (300 nM) for 30 min at 37 °C. The release of EPO activity into supernatants was determined by photometry. Data is expressed as a percentage of the maximal control response (300 nM). * *p <* 0.05 between groups.

*SOCS3* gene expression interference by siRNAs resulted in an evident recovery of eosinophil degranulation after stimulation with either Th2 cytokines (IL-4, IL-5, and IL-13) or PGE_2_ (Figure 5). The recovery percentages were 41, 73, 100, and 100 for PGE_2_, IL-4, IL-5, and IL-13, respectively. Moreover, stimulation with IL-4, IL-5, and IL-13 underwent a significant increase (*p <* 0.05, Figure 5).

### 2.6. JAK-STAT but not MAPK/ERK Pathway Is Affected by SOCS3 Down-Regulation in Eosinophils from Asthmatics

SOCS3 exerts its function by inhibiting activation of several pathways. Therefore, using Western blotting, we evaluated the phosphorylation levels of STAT3 and ERK 1/2, which are JAK/STAT and MAPK/ERK components, respectively. Eosinophils were stimulated with IL-5 for an hour in order to induce SOCS3 expression.

**Figure 6:**
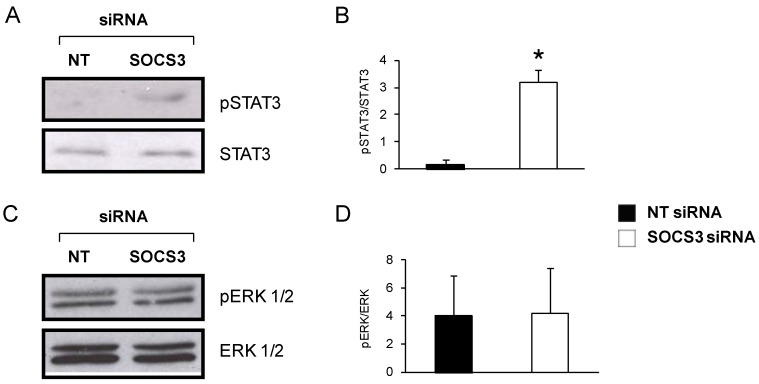
Determination of pSTAT3 and pERK 1/2 protein levels in eosinophils from asthmatic patients. (**A**) Representative pSTAT3 and STAT3 Western blots of blood eosinophils from an asthmatic patient treated with SOCS3 siRNA or NT siRNA for 48 h; (**B**) Densitometric quantification expressed as mean ± SD, *n* = 3. * *p* < 0.05 between groups; (**C**) Eosinophil pERK 1/2 and ERK Western blot after treatment with SOCS3 siRNA or NT siRNA for 48 h; and (**D**) pERK 1/2 and ERK bands were quantified by densitometry. SOCS3 siRNA (white bars, mean ± SD) and NT siRNA (black bars, mean ± SD), *n* = 3.

Interestingly, phospho-STAT3 detection bands were significantly higher and intense after SOCS3 interference by siRNA technology in eosinophils, as depicted in a representative Western blot image in Figure 6A, and further corroborated in Figure 6B by band densitometry (*p* < 0.05; *n* = 6). However, treatment with the non-target siRNA resulted in a complete extinction of any p-STAT3 signal. ERK 1/2 activation was also ascertained, but in this case the phosphorylation status barely changed between eosinophils normally expressing SOCS3 and eosinophils with an impaired SOCS3 expression (Figure 6C,D).

## 3. Discussion

The presence of peripheral blood eosinophilia and activated eosinophils in airway infiltrates are central characteristics in allergic asthma. The clinical importance of eosinophils in asthma relies on the exacerbation frequency in patients, which are traditionally associated with high sputum eosinophil counts and the corresponding decrease in exacerbations when anti-inflammatory therapy is adjusted to maintain low sputum eosinophil percentages [15,16]. In contrast, the relation between blood eosinophilia and asthma exacerbation has received less attention in research. However, Tran *et al.* have recently linked higher blood eosinophil counts with an increase in asthma attacks in asthmatic patients [17]. SOCS3 expression has been positively correlated with asthma severity and serum IgE levels [12]; moreover, a successful animal model has been tested to demonstrate SOCS3 involvement in asthma modulation [18]. Our group has developed an intranasal therapy to deliver a pool of SOCS3 siRNA into the lungs of chronic asthmatic mice, leading to a decrease in lung eosinophilia as well as a significant reduction of AHR and mucous in the airways, which in turn improves chronicity and remodeling [14]. Therefore, we wanted to further assess the importance of SOCS3 in eosinophils from asthmatic patients using siRNA technology. Gene silencing is one rapidly evolving tool in molecular biology, which has the ability to significantly lower the levels of a specific protein, in this case SOCS3, to establish an unequivocal connection between the protein expression and its function in the cell.

Significant effort has been devoted to establish a stable and specific therapeutic adjuvant to effectively introduce siRNA into cells, but in eosinophils these efforts have been unsuccessful. To knock down SOCS3 in eosinophils, we directly cultured siRNA and eosinophils without using viral vectors or a transfection agent, because previous studies reported that eosinophils are able to directly capture the siRNA using the endocitic machinery, which is mainly mediated by dinamin, as demonstrated Goplen *et al.* [19].

In the present study, we confirmed that siRNA could be incorporated into eosinophils and produce a significant suppression of SOCS3. This inhibition is specific, because SOCS1 and SOCS5 mRNA levels are invariably maintained before and after SOCS3 siRNA action in blood eosinophils. This corroborates the notion that siRNA exclusively targets SOCS3 mRNA to produce its degradation and consequently inhibition of SOCS3 protein expression.

*SOCS3* gene silencing has allowed us to overcome a profound immune response study in eosinophils from asthmatic patients. The principal regulatory transcription factors to conduct the polarization into different cell phenotypes were determined: GATA-3, T-bet, and FoxP3. To date, GATA-3 is the only factor described in eosinophils [20], T-bet and FoxP3 transcripts in eosinophils had not been detected until the present work. We decided to evaluate T-bet and FoxP3 expression to better define the immune response type, and even characterized different phenotypes, as has already been done in T lymphocytes [21], macrophages [22], or neutrophils [23]. SOCS3 expression interference led to inhibition of GATA-3 transcripts and, therefore, of the Th2 response. However, the master transcription factor T-bet, which is implicated in Th1 polarization, did not modify its expression significantly after the SOCS3 siRNA treatment in eosinophils. Moreover, the regulatory transcription factor FoxP3 and the principal cytokine associated with this factor, IL-10, were assessed. FoxP3 and IL-10 gene expressions were inhibited by *SOCS3* gene silencing in eosinophils.

SOCS3 participation in Th2 responses has been described in several research articles [11,24,25]; in contrast, SOCS3 implication in the establishment of the regulatory phenotype is controversial. For example, SOCS3 reduction in T lymphocytes causes an increase in IL-10 and CTLA-4 levels [26], but in dendritic cells and in synovial fluids from arthritic processes, SOCS3 expression is positively associated with IL-10 production [27,28].

Migration, adhesion and degranulation are three important actions during the eosinophil lifespan, which have been tested. Inhibition of *SOCS3* gene expression by siRNA technology has been proven to reduce eosinophil migration towards an IL-5, eotaxin or fMLP gradient stimuli due to a decrease in CCR3 expression on the eosinophil surface. IL-5 and eotaxin work synergistically to potentiate CCR3 expression and boost the receptor aggregation [29], through a clathrin-dependent reduction of the internalization rate [30]. Interleukin-5 is also an eosinophil adhesion inductor to the vascular endothelium [31]; besides, IL-13 has been described as an active player in the beginning of the adhesive process [32]. Intriguing, SOCS3 expression abrogation in blood eosinophils from asthmatic patients results in a loss of adhesion capacity in the presence of Th2 interleukins, such as IL-5 and IL-13. Impaired adhesion is also observed when blood eosinophils are stimulated with PGE_2_. Sturn *et al.* defined PGE_2_ as an inhibitor of the eosinophil trafficking acting through their EP_2_ receptors [33]. In fact, there are many open research lines focusing on the importance of arachidonic acid metabolites in the regulation of cellular trafficking [34,35], meaning IL-5, IL-13, and PGE_2_ probably act through SOCS3 to improve eosinophil adhesion.

We wanted to further investigate the role of SOCS3 in adhesion function enhancement, and several adhesion molecules were measured by flow cytometry in blood eosinophils either treated with SOCS3 siRNA or control NT siRNA. Integrins VLA-4 and LFA-1 were drastically reduced when eosinophils were incubated with SOCS3 siRNA. These 2 integrins are critical in the eosinophil recruitment and subsequent activation at inflammatory regions in allergic asthma [36]. In particular, IL-5 and GM-CSF stimulation in purified eosinophils from human blood increases LFA-1 expression after the first allergen contact [37]. Therefore, SOCS3 could control the induction of adhesion molecules in eosinophils through the NF-κB factor activation [38,39], widely associated in the literature with adhesion function [40].

Once eosinophils reach the inflammatory regions, degranulation becomes one of the key effector functions. The most aggressive degranulation is cytolytic degranulation in which eosinophils release all their granule cationic content. In this study, cytolytic degranulation has been induced in eosinophils by C5a addition to the cultures [41]. Eosinophils deficient in SOCS3 protein due to gene interference that were later stimulated with Th2 cytokines, such as IL-4, IL-5, and IL-13, had an increased degranulation in comparison with those eosinophils treated with NT siRNA. The augmented degranulation in SOCS3 siRNA treated eosinophils entails cell death before they reach the target tissue, in this case the lung, and the consequent content liberation within the bloodstream. Therefore, as we demonstrated in the mouse model of allergic asthma treated with SOCS3 siRNA intranassally [14], SOCS3 up-regulated expression in asthma has to be controlled, in this case by gene silencing in order to restore SOCS3 homeostatic levels. Thus, instead of downregulation of SOCS3 expression in blood human eosinophils, it would be more reasonable in terms of therapy to control SOCS3 levels to avoid a massive degranulation.

The effect of SOCS3 on two signal transduction pathways, JAK/STAT and RAS/ERK, both tightly controlled by the suppressor among other mechanisms, has also been evaluated through an analysis of their principal intermediates using Western blotting. On one hand, the JAK/STAT pathway remains constitutively activated when SOCS3 expression is almost suppressed in purified eosinophils through phospho STAT3 augmentation, as has been previously reported in other cellular types [42]. On the other hand, *SOCS3* gene silencing in blood eosinophils from asthmatics does not cause any alteration in ERK1/2 phosphorylation, an intermediate of the RAS/ERK pathway. However, RAS/ERK signaling activation in blood eosinophils seems to be directly implicated in their migration and degranulation when eosinophils are stimulated with a strong chemoattractant, such as eotaxins, instead of the stimuli used in the present study, IL-5 and GM-CSF [43,44].

On account of all these results, it seems that after SOCS3 downregulation by siRNAs, the eosinophil acquires a less Th2 phenotype, due to an inhibition of GATA-3, FoxP3 and IL-10 transcription, which is directly connected with a reduced migration and adhesion that affects the eosinophil capacity to extravasate into the lungs. Hence, eosinophils with this “regulatory” phenotype would be able to exert a new role in asthma pathogenesis controlling the inflammatory process.

## 4. Experimental Section

### 4.1. Subjects

Thirty-six subjects with asthma were recruited from the Allergy Department of the Fundación Jiménez Díaz hospital. The patients’ clinical characteristics are shown in Table 1.

**Table 1:** Clinical characteristics of asthma patients.

Items	Subjects
*n*	36
Age (years) *	46 (20–86)
Male (%)	11 (28.2)
Atopy (%)	29 (74.2)
FEV_1_ (%) *	100 (73–138)
FEV_1_/FVC (%) *	79 (62–100.4)
FeNO ppb *	31 (8.5–230)
Peripheral eosinophilia (%) *	4.7 (1.5–19.1)

FVC, forced vital capacity; FEV_1_, forced expiratory volume in the first second; ppb, parts per billion; FeNO, fraction of exhaled nitric oxide. * Median (range).

All subjects with asthma had a consistent history of the disease and objective evidence of asthma (as defined by the American Thoracic Society) [45] for at least 6 months. These patients either showed a greater than 12% improvement in FEV_1_, 10 min after administration of 500 μg of inhaled terbutaline, or had methacholine airway hyperresponsiveness (PC_20_ methacholine <16 mg/mL). Most of the subjects reported mild persistent disease [46] and were clinically stable; 29 were atopic and none had a history of respiratory infection over the 6-week period preceding the study. We included both atopic and nonatopic patients in the asthmatic group since no differences in the parameters assessed for both sets of patients had been observed previously. For patients who were receiving inhaled corticosteroids, the drugs were withdrawn for at least 2 weeks before the blood samples were taken. No patient was receiving oral corticosteroids (for at least 6 months prior to the study), leukotriene receptor antagonists, aspirin, or any other cyclooxygenase inhibitor.

This study was conducted according to Good Clinical Practice (GCP) standards and the Helsinki Declaration, and it was approved by the Fundacion Jiménez Díaz Ethics Committee (CEIC). All patients provided written informed consent to participate in the study and consented to the extraction of peripheral blood.

### 4.2. Recombinant Proteins and Reagents

Recombinant human IL-5, IL-13, and GM-CSF were purchased from R&D System (Minneapolis, MN, USA), IL-4 from Bender MedSystem, (Vienna, Austria), and PGE_2_ from Cayman Chemical Company (Ann Arbor, MI, USA). Abs against phospho-STAT3 (Ser^727^), STAT3, phosphor-ERK1/2, ERK1/2, SOCS3, and β-actin were from Cell Signaling Technology (Beverly, MA, USA). Anti-human CD54 PE, LFA-1 FITC, CD106 FITC, VLA-4 PE, CD49b FITC, and CCR3 FITC were all flow cytometry antibodies purchased from Becton Dickison (Franklin Lakes, NJ, USA).

### 4.3. Eosinophil Isolation

Eosinophils were purified from the peripheral blood of healthy control and patient donors using a 2-step procedure as previously described [30]. First, the polymorfonuclear fraction was obtained by density gradient centrifugation using Lymphoprep^®^ (Rafer, Zaragoza, Spain), followed by lysis of the red cells with an ammonium chloride solution (155 mM NH_4_Cl, 10 mM KHCO_3_, and 0.1 mM EDTA). The second step involved removal of residual cells from the polymorphonuclear cell fraction. For eosinophil purification, CD2, CD3, CD14, CD16, CD19, CD20, CD36, CD56, CD123, and glycophorin A positive cells were discarded using the magnetic bead separation technique, as described by the manufacturer (EasySep^®^ StemCell Technologies, Vancouver, Canada). Cells were stained with fluorochrome-conjugated anti-CCR3 (CCR3-FITC) and anti-CD16 (CD16-PE) antibodies. Eosinophil (CCR3^+^CD16^−^ cells) purity was routinely >98% as measured by flow cytometry.

### 4.4. Eosinophil Culture

Purified eosinophils were suspended in RMPI-1640 medium (Sigma-Aldrich Corp., Detroit, MI, USA), supplemented with 0.1 mM nonessential amino acids, 100 U/mL penicillin, 100 µg/mL streptomycin, 10 mM HEPES, 2 mM l-glutamine, and 10% (*v*/*v*) fetal bovine serum (Lonza Corp., Basel, Switzerland). The culture medium was also supplemented with a cocktail of IL-5 (10 ng/mL)/GM-CSF (10 ng/mL). The culture cells were maintained at 37 °C in a 5% CO_2_ atmosphere.

An equimolar mix of 3 different sequences of SOCS3 siRNA (s17190: sense 5'-AGAAGAGCCUAUUACAUCUTT-3', antisense 5'-AGAUGUAAUAGGCUCUUCUGG-3'; s17191: sense 5'-GCACCUUUCUGAUCCGCATT-3', antisense 5'-UCGCGGAUCAGAAAGGUCCG-3'; s17189: sense 5'-UGAUUUGGUUUAAACCUGATT-3', antisense 5'-UCAGGUUUAAACCAAAUCAAA-3') or negative control siRNA (non-target) were added to the culture (150 nm), all of which were purchased from Ambion. Eosinophils were incubated for 48 h to perform functional experiments or lysed in Trizol Reagent^®^ (Invitrogen, Carlsbad, CA, USA). Alternatively, cells were briefly centrifuged (1 min, 12,000 rpm), suspended in sample buffer 1.5× (10^6^ cells in 50 μL), and boiled for 5 min.

### 4.5. RNA Isolation, RT-PCR, and TaqMan Gene Expression Assays

Total RNA was isolated from purified human eosinophils according to TRizol^®^ protocol. One microgram of RNA was reverse-transcribed to cDNA using a high-capacity cDNA Reverse Transcription Kit (Applied Biosystems, Warrington, UK).

Quantitative real-time PCR was performed on a 7500 Real-Time PCR system (Applied Biosystems). TaqMan gene expression master mix and TaqMan gene expression assay probes (SOCS3, SOCS1, SOCS5, GATA3, T-bet, IL-10, FoxP3, 18S) were from Applied Biosystems and were used for qRT-PCR to determine mRNA levels. Messenger RNA expression was calculated for each sample using the cycle threshold (*C*_t_) value. The relative gene expression was calculated as follows: 2^−ΔΔ*C*t^, where ΔΔ*C*_t_ = Δ*C*_target gene_ − Δ*C*_18s_ [47].

### 4.6. Fluorescence Microscopy

Purified eosinophils from asthmatic patients were fixed in 4% paraformaldehyde and suspended in quenching solution (PBS and 50 mM ammonium chloride). Then, cells were immersed in blocking solution (PBS/1% BSA/0.5% saponin) and washed twice with a permeabilization solution (0.1 M glycine, 0.5% saponin, 0.1 M HEPES, 1% BSA) before incubation with a mouse anti-human SOCS3 antibody (Santa Cruz Biotechnology, Dallas, TX, USA). After, eosinophils were incubated with a secondary antibody labeled with Alexa 647 fluorochrome (Molecular Probes, Life Technologies, Carlsbad, CA, USA). Finally, to visualize eosinophil nuclei, cells were stained with DAPI 300 nM (Molecular Probes, Life Technologies) and included in a solution to protect fluorescence (Prolong, Molecular Probes, Life Technologies). Eosinophils were observed using fluorescence microscopy (Nikon Eclipse TE2000S, Melville, NY, USA). Images were processed using Image J software (National Institutes of Health, Bethesda, MD, USA).

### 4.7. Eosinophil Migration

Cell-culture inserts with a 5-μm pore (Millipore, Billerica, MA, USA) were placed in a 24-culture plate well. The lower wells were filled with 600 μL of buffer alone or buffer containing 10 ng/mL of recombinant IL-5, or 50 ng/mL of eotaxin, or 1 µM of fMLP. Then, aliquots of 2 × 10^6^ eosinophils resuspended in 250 μL of RPMI 1640 without phenol-red, 10% FBS, IL-5, and GM-CSF (10 ng/mL) were added on the top filter membrane. The plates were incubated at 37 °C for 90 min. Migrated cells in the lower chambers were collected and counted by flow cytometry for 3 min (FACS CANTO II, BD, Franklin Lakes, NJ, USA). Results are expressed as incresase/decrease percentage in migration relative to migration towards the control medium. The optimal doses of stimuli were determined previously performing dose-response curves.

### 4.8. Eosinophil Adhesion

Adhesion assay was performed as previously described [48,49], where EPO activity are used as a marker of eosinophil adhesion. Ninty-six-well plates were pre-coated with a fibronectin solution (20 µg/mL in PBS) overnight at 4 °C, following 0.1% BSA (*w*/*v*) incubation for 60 min at 37 °C. Human purified eosinophils treated with SOCS3 siRNA or scrambled siRNA were stimulated with IL-5 or IL-4 (10 ng/mL; Bender MedSystem, Vienna, Austria) or IL-13 (10 ng/mL R&D System) or PGE_2_ (10^−6^ M) for 1 h. Then, eosinophils were added to fibronectin-coated wells in a volume of 50 µL of medium without phenol red (7 × 10^4^ cells/mL) to the coated wells. Eosinophil adhesion to wells was allowed for 30 min at 37 °C, 5% CO_2_, plate were washed twice with PBS to discard non-adhered cells. Wells were re-filled with 50 µL of medium, and a standard curve was achieved by addition of varying concentrations of the original cell suspension within empty wells. Eosinophil adhesion was calculated by measuring residual eosinophils peroxidase (EPO) activity of adherent cells. EPO substrate (1 mM H_2_O_2_, 1 mM o-Phenylenediamine, and 0.1% Triton X-100 in Tris Buffer, pH 8.0) was then added to all the wells. After 30 min-incubation at room temperature, 25 µL of 4 M H_2_SO_4_ were used to stop the reaction. Microplates were analyzed on a bench reader at 490 nm. Adherence was calculated by comparing absorbance of unknowns towards that of the standard curve. All conditions were assayed in triplicate in at least 6 independent experiments.

### 4.9. Eosinophil Degranulation

To determine the release of eosinophil peroxidase (EPO) from purified eosinophils from asthmatic patients, cells were resuspended in assay buffer (PBS, 0.1% BSA, 10 nm HEPES, 10 nm Glucose; pH 7.4) at 1 × 10^6^ cells/mL, mixed with cytochalasin B (10 μg/mL), and 50-μL aliquots were loaded into the wells of a 96-well microplate. Cells were stimulated with 20 μL of C5a (300 nM) for 20 min at 37 °C. Thereafter, 60 μL of H_2_O_2_ (1 mM) were added to each well to start the peroxidase reaction. To detect the reaction, 70 μL of 2.8 mM tetramethylbenzidine was used. Following incubation for 1 min at room temperature, the peroxidase reaction and the color development were stopped with 4 M acetic acid. Microplates were analyzed on a bench reader at a wavelength of 630 nm. Data were expressed as the percentage of the maximal control response (C5a at 300 nM).

### 4.10. Flow Cytometry

Eosinophils (1.5 × 10^5^) were resuspended in PBS/iFBS and stained with different antibodies (anti-human CCR3 FICT, CD54 PE, LFA-1 FITC, CD106 FITC, VLA-4 PE, CD49b FITC) for 20 min on ice. Then, the cells were washed twice with PBS/iFBS and analyzed by flow cytometry in a FACS CANTO II cytometer (BD).

### 4.11. Immunoblot

Forty microliters of purified human eosinophil lysate was resolved on SDS-PAGE and probed with specific Abs at the appropriate dilution (phospho-STAT3 1:500, STAT3 1:1000, phospho-ERK1/2 1:2000, ERK 1/2 1:1000, SOCS3 1:500, β-Actin 1:2000). Chemiluminescent protein bands were detected by an ECL detection system (Amersham Biosciences, GE Healthcare, Buckinghamshire, UK) according to the manufacturer’s protocol.

## 5. Conclusions

*SOCS3* gene silencing in eosinophils purified from peripheral blood from asthmatic patients leads to an inhibition in the migration and adhesion capacities of eosinophils, which avoids extravasation to the inflammatory focus. Resolution of eosinophilic inflammation is a critical goal for asthma therapy, and the promotion of a phenotype switch in eosinophils through SOCS3 down-regulation could be a possible strategy to achieve this goal.
